# Temporal plus epilepsy: Anatomo-electroclinical subtypes

**Published:** 2016-07-06

**Authors:** René Andrade-Machado, Vanessa Benjumea-Cuartas

**Affiliations:** 1 Department of Epilepsy, National Institute of Neurology, CES University, Medellín, Colombia; 2 National Institute of Neurology, CES University, Medellín, Colombia

**Keywords:** Temporal Lobe Epilepsy, Insular Cortex, Drug Resistant; Neural Networks; Intracranial Electroencephalography; Positron Emission Tomography

## Abstract

**Background:** Mesial temporal lobe epilepsy (TLE) is a remediable epileptic syndrome. About 40% of patients continue to have seizures after standard temporal lobectomy. It has been suggested that some of these patients could actually suffer from a more complex epileptogenic network. Because a few papers have been dedicated to this topic, we decided to write an article updating this theme.

**Methods:** We performed a literature search using the following terminology: “temporal plus epilepsy and networks,” “temporal plus epilepsy,” “orbito-temporal epilepsy,” “temporo-insular epilepsy,” “temporo-parieto-occipital (TPO) epilepsy,” “parieto-temporal epilepsy,” “intracortical evoked potential and temporal plus epilepsy,” “temporal lobe connectivity and epilepsy,” “intracortical evoked potential and epilepsy surgery,” “role of extratemporal structures in TLE,” “surgical failure after temporal lobectomy,” “Diffusion tensor imaging (DTI) and temporal epilepsy,” and “positron emission tomography (PET) in temporal plus lobe epilepsy” in the existing PubMed databases. We searched only English and Spanish literature. Only papers that fit with the above-mentioned descriptors were included as part of the evidence. Other articles were used to reference some aspects of the temporal plus epilepsy.

**Results:** A total of 48 papers from 2334 were revised. The most frequently reported auras in these groups of patients are gustatory hallucinations, vestibular illusions, laryngeal and throat constriction, atypical distribution of somatosensory symptoms (perioral and hands, bilaterally hands paresthesias, trunk and other). The most common signs are tonic posturing, hemifacial twist, and frequent bilateral clonic movements. Interictal electroencephalographic (EEG) patterns exhibit regional and frequently bilateral spikes and/or slow waves. The first ictal electrographic change is mostly regional. It is important to note that the evidence is supported by case series or case reports. Thus, most of the data presented could represent the features on these cases and not actually the totality of the iceberg.

**Conclusion:** Temporal plus epilepsy is a diagnosis that can be done only after the invasive recordings have been analyzed but an adequate suspicion may arise based on clinical, EEG and imaging data.

## Introduction

**Figure 1:**
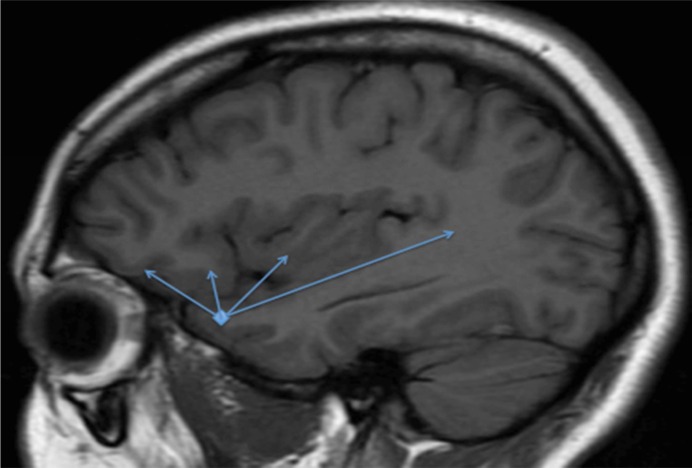
Sagittal magnetic resonance imaging (MRI) of the head

Temporal lobe epilepsy (TLE) with hippocampal sclerosis may have different semiological, ictal and interictal electroencephalographic (EEG), and histopathological features.^1^^-^^3^ Although more than 60% of patients remained seizure-free after temporal lobectomy, recurrence from an extratemporal, contralateral or neocortical focus are a commonly reported causes of surgical failure.^4^^-^^11^ Alternately, it has been suggested that some patients with surgically refractory TLE could actually suffer from a more complex epileptogenic network that can encompass the temporal lobe and brain regions to which it is closely related. Ryvlin and Kahane^6^ recently introduced the term temporal plus epilepsy (TL+) to designate this form of multilobar epilepsy: an epilepsy in which primary temporal lobe epileptogenic zone is extended to neighbored regions, such as the insula, the suprasylvian operculum, the orbitofrontal cortex (OFC), and the temporo-parieto-occipital (TPO) junction (Figure 1). Until now this, type of epilepsy is only correctly identified using invasive recordings. Because a few papers have been dedicated to this theme and the growing scientific evidence supporting the existence of TL+ epilepsy and the importance of recognize this type of epilepsy to avoid surgical failures, we decided to write an article focused on this topic. 

The arrows (from anterior to posterior part of the head) represent some common origins in temporal plus epilepsy (OF, operculum, insular cortex, and TPO junction).

## Materials and Methods

We performed a literature search in the PubMed, Medline, and Cochrane databases with the aim of determining the electroclinical and imaging features of temporal plus epilepsy and the white matter (WM) connections underlying the epileptogenic networks studying through intracortical evoked potentials or diffusion tensor imaging (DTI) techniques.

**Figure 2:**
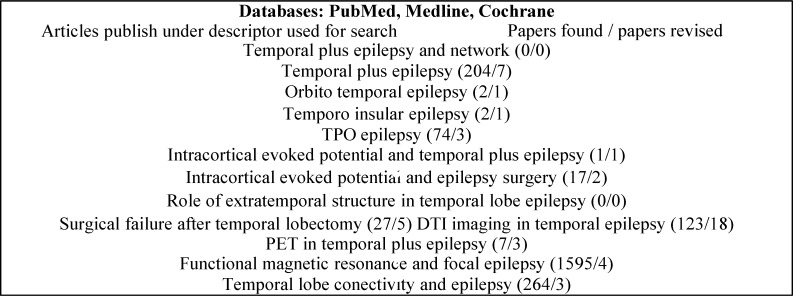
Flow of information through the searching of review process

DTI: Diffusion tensor imaging; PET: Positron emission tomography; TPO: Temporo-parieto-occipital

Published case series, case reports, original articles and literature reviews of the temporal plus epilepsy or the epileptogenic networks in TLE were identified using the search terms “temporal plus epilepsy and networks,” “temporal plus epilepsy,” “orbito-temporal epilepsy,” “temporo-insular epilepsy,” “TPO epilepsy,” “parieto-temporal epilepsy,” “intracortical evoked potential and temporal plus epilepsy,” “temporal lobe connectivity and epilepsy,” “intracortical evoked potential and epilepsy surgery,” “role of extratemporal structures in TLE,” “surgical failure after temporal lobectomy,” “DTI imaging and temporal epilepsy,” and “positron emission tomography (PET) in temporal plus lobe epilepsy” in the existing databases. We searched only English and Spanish literature (Figure 2) (Table 1).

**Table 1:** Descriptors, papers found and revised, first authors and the title for each search

**Descriptors**	**Papers found/papers revised**	**Author**	**Publication**
Temporal plus epilepsy and networks	0		
Temporal plus epilepsy	204/7	Thompson et al.^12^	Auditory aura in frontal opercular epilepsy: sounds from afar
		Kahane et al.^13^	The concept of temporal “plus” epilepsy
		Guedj et al.^14^	18FDG-PET in different subtypes of TLE: SEEG validation and predictive value
		Rathore et al.^15^	The utility of 18F-FDG-PET in epilepsy surgery
		Zhu et al.^16^	Temporal plus epilepsies: electrophysiology studied with interictal MEG and intracranial video-EEG monitoring
		Harroud et al.^4^	TLE surgery failures: a review
		Barba et al.^5^	Ictal clinical and scalp-EEG findings differentiating temporal lobe epilepsies from temporal “plus” epilepsies
		Ryvlin and Kahane.^6^	The hidden causes of surgery-resistant TLE: extratemporal or temporal plus?
Orbito temporal epilepsy	2/1	Mesulam and Mufson^17^	Insula of the old world monkey. I. Architectonics in the insulo-orbito-temporal component of the paralimbic brain
Temporo-insular epilepsy	2/1	de Maeseneire et al.^18^	Musical hallucinations as a presenting manifestation of a left temporo-insular glioma
TPO epilepsy	74/3	Marossero et al.^19^	SEEG and surgery in partial epilepsy with TPO foci
		Williamson et al.^11^	Parietal lobe epilepsy: diagnostic considerations and results of surgery
		Palmini et al.	Occipitotemporal epilepsies: evaluation of selected patients requiring depth electrodes studies and rationale for surgical approaches
Intracortical evoked potential and temporal plus epilepsy	1/1	Enatsu et al.^20^	Connections of the limbic network: A CCEPs study
Intracortical evoked potential and epilepsy surgery	17/2	Almashaikhi et al.^21^	Functional connectivity of insular efferences
		Catenoix et al.^22^	Evoked potential study of hippocampal efferent projections in the human brain
Role of extratemporal structures in TLE	0		
Surgical failure after temporal lobectomy	27/5	Blauwblomme et al.^23^	Prognostic value of insular lobe involvement in TLE: a SEEG study
		Ramos et al.^7^	Failure of temporal lobe resection for epilepsy in patients with mesial temporal sclerosis: results and treatment options
		Jeha et al.^8^	Predictors of outcome after temporal lobectomy for the treatment of intractable epilepsy
		Janszky et al.^9^	Failed surgery for TLE: predictors of long-term seizure-free course
DTI imaging and temporal epilepsy	123/18	Labate et al.^24^	WM abnormalities differentiate severe from benign TLE
		Catani and Thiebaut de Schotten^25^	A DTI tractography atlas for virtual *in vivo* dissections
		Kemmotsu et al.^26^	Frontolimbic brain networks predict depressive symptoms in TLE
		Liacu et al.^27^	DTI tractography parameters of limbic system bundles in TLE patients
		Bhardwaj et al.^28^	Diffusion tensor tractography detection of functional pathway for the spread of epileptiform activity between temporal lobe and Rolandic region
PET in temporal plus epilepsy	7/3	Guedj et al.^14^	18FDG-PET in different subtypes of TLE: SEEG validation and predictive value
		Rathore et al.^15^	The utility of 18F-FDG-PET in epilepsy surgery
		Boling et al.^29^	FDG-PET imaging for the diagnosis of MTLE
Functional MR and focal epilepsy	1595/4	Avesani et al.^30^	EEG-fMRI evaluation of patients with mesial temporal lobe sclerosis
		Kaiboriboon et al.^31^	Interictal MEG/MSI in intractable MTLE: spike yield and characterization
		Al-Asmi et al.^32^	fMRI activation in continuous and spike-triggered EEG-fMRI studies of epileptic spikes
		Manganotti et al.^33^	Continuous EEG-fMRI in patients with partial epilepsy and focal interictal slow-wave discharges on EEG
Temporal lobe connectivity and epilepsy	264/3	Haneef et al.^34^	Functional connectivity of hippocampal networks in TLE
		Antony et al.^35^	Functional connectivity estimated from intracranial EEG predicts surgical outcome in intractable TLE
		Kemmotsu et al.^36^	Alterations in functional connectivity between the hippocampus and prefrontal cortex as a correlate of depressive symptoms in TLE

TLE: Temporal lobe epilepsy; TPO: Temporo-parieto-occipital; 18F-FDG-PET: 18F-fluorodeoxyglucose-positron emission tomography; MEG: Magnetoencephalography; EEG: Electroencephalographic; CCEP: Cortico-cortical evoked potentials; DTI: Diffusion tensor imaging; WM: White matter; fMRI: Functional magnetic resonance imaging; MSI: Magnetic source imaging

Only papers that fit with the above-mentioned descriptors were included as part of the evidence if it’s described the symptomatology, electrographic and imaging features and intracortical evoked potentials in patients with well documented TL+ epilepsy. TL+ epilepsy was considered well documented if in the section “patients and methods” one of the following criteria appeared well described:

1. Seizure freedom after 2 years of surgery in a patient with a wide resection that included temporal lobe structures and one of the following: the insular lobe, OFC, TPO junction or suprasylvian cortices
2. The stereo-electrocorticography (ECoG) showed an ictal onset zone involving insular, OF, TPO junction or suprasylvian cortices during temporal lobe seizures.

From our literature review, we organized the present article into sections detailing the temporal plus network and anatomy, semiology of seizures, features from scalp recording, PET scan, and evoked potentials.

***Temporal plus networks and anatomy***

Temporal plus neural network encompass different interconnections among many cortical and subcortical structures (parietal lobe, temporal lobe, occipital lobe, temporo-parietal-occipital junction, insular lobe, perisylvian cortex, OF cortical areas, and the cingulate gyrus). Primary and/or secondary epileptogenesis in this broad circuitry explain the different patterns of seizure semiology, interictal or ictal EEG patterns, and hypometabolism in PET images. The knowledge of this circuits can be helpful to understand the temporal plus concept, the planning of invasive studies and finally to decide which should be the epileptogenic zone. Three methods have been of helpful to understand the connectivity among these cortical areas: intracortical evoked potentials, functional resonance images, and post-mortem anatomy dissection. These studies have identified different neural networks: limbic, temporoparietal, TPO, OF, perisylvian, and opercular networks.^24^^-^^28^^,^^37^

***Temporal plus epilepsy: anatomic correlation***

The limbic system as a part of the network in temporal lobe epilepsies: Papez^8^ proposed a system involved in emotion and episodic memory, which is composed of the hypothalamus, hippocampus, mammillary bodies, thalamus, cingulate gyrus, parahippocampal gyrus (PHG), and the entorhinal cortex (EC). Thereafter, Maclean^39^ introduced the neurophysiological and neuroanatomical concept of the “limbic system.” He added the OFC and amygdala, and named this group of structures the “limbic system.”

Cortico-cortical evoked potentials (CCEP) have demonstrated that a functional connectivity among various components of the human limbic network subserves as a model of epileptogenic network.^20^ Data taken from cortical EEG recordings and from right anterior hippocampal stimulation during invasive recordings in patients with focal refractory epilepsy shows that it elicits prominent CCEPs responses in the ipsilateral medial and lateral temporal structures, operculum, medial and lateral OF, medial and lateral prefrontal cortex, supplementary motor cortex (SMA), pre-SMA, anterior cingulated gyrus, and insular cortex.^20^^,^^34^^-^^36^

The above-mentioned data have some possible implications for the interpretation of the semiology and EEG recordings in patients with suspected TL+ epilepsy. There are strong intra- and interhemispheric connections from these cortical areas to the temporal lobe. This broad intrahemispheric connection can explain the difficulty of interpreting scalp or invasive EEG in epilepsies related with the above-mentioned neural networks.^20^

TPO junction: TPO junction is a complex region of the brain involved in several crucial high-level functions such as language, visuospatial recognition, writing, reading, symbol processing, calculation, self-processing, working memory, musical memory, and face and object recognition.^40^ The seizure involvement of this associative cortex gives arise some of the typical symptoms described in TL+ epilepsy (such as auditive, vestibular, and visual auras). The special functional relations in between these cortices could explain emotional changes and postictal amnesia.

At the subcortical level, the WM of the TPO junction represents a crucial node of intralobar and interlobar connectivity that could explain the above-mentioned symptomatology. In fact, the activation of cortical neurons or their axons in TPO junction produces functional changes at both local and distant brain hubs, and the modifications differ based on specific patterns of connectivity.^25^^,^^41^^-^^45^

The insular lobe: The insular lobe has functional connectivity with the hippocampus, EC, frontal, temporal and parietal opercula, temporal pole, lateral temporal neocortex, pre-central and post-central regions, perisylvian region, OFC, and amygdala. Most of the connections are reciprocal.^17^^,^^21^ These connections explain the difficulty to assess insular seizures without invasive recording and the frequency of misleading electrographic patterns when insular seizures are evaluated with scalp electrodes.

The cingulated gyrus: The cingulated gyrus has strong and bidirectional connections with the temporal lobe. The efferent fibers from cingulated cortex reach the hippocampus by the final part of nauta circuitry, throughout the parahippocampal cortex, subiculum, and dentate gyrus.^28^^,^^34^^,^^35^ This circuitry can explain why cingulated seizures can easily mimic temporal seizures.

***WM fibers as skeleton of TL+ epilepsy***

1. Uncinate fasciculus: this pathway connects the anterior part of the temporal pole, the uncus, amygdala and hippocampal gyrus to the orbital and polar frontal cortex^40^
2. Inferior longitudinal fasciculus connects the anterior part of the temporal lobe to the occipital lobe. Recent DTI studies have demonstrated the existence of both a direct and indirect pathway^40^
3. Inferior occipitofrontal fasciculus: this tract comes from the occipital lobe and postero-lateral temporal areas to the OF and dorso-lateral prefrontal cortices via the anterior floor of the external capsule. The Inferior occipitofrontal fasciculus terminations appears within the superior parietal lobe, superior occipital gyrus, medial occipital gyrus and inferior occipital gyrus, and within the temporo-basal region^40^
4. Superior longitudinal fasciculus (SLF) (or arcuate fasciculus) is a fiber tract stemming from the caudal part of the posterior and superior temporal cortex (mainly Wernicke’s area) that arches around the insula and projects forward to end within the frontal lobe (mainly prefrontal and premotor gyri, especially Broca’s area). Three segments of the perisylvian SLF have been identified: (1) anterior segment, connecting the supramarginal gyrus and superior temporal gyrus with the precentral gyrus, (2) posterior segment, connecting the posterior portion of the middle temporal gyrus with the angular gyrus, and (3) long segment of the arcuate fasciculus that connects the middle and inferior temporal gyri with the precentral gyrus and posterior portion of the inferior and middle frontal gyri.^46^

***From the anatomy to the clinical features in TL+ epilepsy***

According to Barba et al.^5^ and other series of cases and case reports, we can describe the semiology of TL+ epilepsy as following:

Auras: The most frequently reported auras in these groups of patients are gustatory hallucinations and vestibular illusions. Although other different types of auras (emotional, psychic, visual, auditive, and vestibular) can be found. Gustatory auras are associated with an ictal onset zone in temporal insular subgroup, and vestibular auras in the TPO subgroup.^5^^,^^11^ Typical auditory auras begin with a non-lateralized or lateralized auditory aura, described as a distortion of sounds (“things sound weird”), accompanied by a feeling of anxiety or as if his/her hearing was “muffled,” associated with an indescribable feeling of an impending seizure. These could be triggered by music and specifically by a sudden change in musical rhythm but also can occur spontaneously. This type of aura can be associated with an ictal onset zone in inferior perisylvian cortex but can be also found in frontal opercular region.^5^^,^^12^ TL + epilepsies less frequently presented an ability to warn at seizure onset, and to report abdominal aura.^5^

Motor manifestations in TL+ epilepsy: The patients suffering from TL+ epilepsies more frequently exhibit versive manifestations of the eyes and/or head, more especially contraversive motion of the head and/or eyes. Other signs found in TL+ epilepsies were piloerection and ipsilateral tonic motor signs. It is important to know that gestural automatisms are less associated with TL+ epilepsy. When secondary generalized seizures appear, a phase of tonic contraction of the hemiface with eye deviation occurred, with an initial extension of the arm on generalization. Furthermore, varied hyperkinetic motor movements and vocalization would follow the initial seizure onset.^10^^,^^11^^,^^19^

Consciousness: Consciousness was impaired in almost all cases in some point during seizures.^5^

Postictal phase: Postictal phase related to temporal epilepsy, postictal amnesia TL+ epilepsy is not frequently found in the postictal phase. Moreover, dysphoric signs are more frequently found in patients with TL+ epilepsy compared to those with TLE. Aphasia for 1-2 minutes or transient oromotor dysfunction (inability to speak) were reported and indicated opercular compromise.^5^^,^^12^^,^^40^

***Role of other cortical structures in temporal plus epilepsy***

Insula: Penfield and Flanigin^47^ had already observed that seizures arising from the insula could mimic temporal lobe seizures. In a series of 21 patients with atypical TLE reported by Isnard^48^ two patients showed stereoelectroencephalography (SEEG) evidence of spontaneous insular seizures, and both achieved a poor outcome (Engel class IV) following temporal lobectomy only.

The contribution of noninvasive investigations in the detection of insular epilepsy masquerading as or concomitant to TLE is limited.^23^ The most useful technique is magnetic resonance imaging (MRI), as visualization of an insular lesion strongly supports the likelihood of insular involvement. Unfortunately, insular epilepsy is commonly non-lesional. Scalp EEG is of limited utility. Single-photon emission computed tomography and PET scans may reveal changes in the insular lobe, but their specificity is low. There is some promising evidence that magnetoencephalography (MEG) can be useful in localizing insular epileptogenic foci, but further studies are needed to assess its true clinical utility. In the absence of a visualized lesion on MRI, intracranial recording with insular sampling is necessary.^23^^,^^48^ This is particularly true for those patients with TLE symptoms who also show “atypical” features such as occurrence at onset of somatosensory (e.g., laryngeal discomfort, throat constriction, limb paresthesias specifically distal hand paresthesia, any combination of perioral and hand paresthesia, trunk and distal hand paresthesia or bilateral sensorial symptoms) or motor symptoms (e.g., arm elevation, trashing, or pedaling).^18^^,^^23^^,^^48^^,^^49^

In an attempt to improve the outcome of TLE surgery, Penfield and Flanigin^47^ were the first to perform insular resection, when residual epileptiform activity in the insula was recorded after removal of the temporal lobe.

However, this approach was abandoned after Silfvenius et al.^50^ reported that this method did not benefit seizure control while significantly increased morbidity from 3 to 21% (mainly hemiparesis). It is of interest that patients in the same study by Silfvenius et al.^50^ who underwent reoperation and had an insulectomy had a rate of unsatisfactory outcome of only 46% compared to 83% in patients who did not have insulectomy, suggesting that the insula could actually play a role in the surgical failure of some patients with TLE. Fortunately, several publications have recently shown that insular epilepsy surgery can be both safe and beneficial with modern neurosurgical techniques.^51^^-^^56^

OF cortex: OF seizures are characterized by integrated gestural motor behavior, distal stereotypes and fearful behavior.^56^

After reviewing case reports, some common features of OF-temporal epilepsy can be summarized as follows: lack of aura or nonspecific auras, autonomic changes, behavioral arrest and/or impaired awareness, complex motor and “hypermotor” activity, vocalization, oculocephalic deviation, olfactory or gustatory hallucinations and occasionally secondary generalization. Occurrence in clusters, nocturnal preponderance, relatively short duration and brief, if any, postictal confusion are also observed.^56^^-^^60^ This semiology can be summarized as three “recognizable seizure patterns:”^57^ (1) Olfactory auras accompanied or not by gustatory auras, autonomic changes, oroalimentary and/or gestural automatisms and “thymic alterations,” (2) autonomic seizures,^57^ (3) the so-called “hypermotor seizures.” These hypermotor movements may appear violent, for example, thrashing, bicycling, kicking, frenetic striking or flailing of limbs and other rather peculiar motor behaviors. As it can be seen epileptogenic foci within the OF-temporal region can give rise to seizures, which are electro clinically indistinguishable from temporal lobe seizures given the widespread connections between the limbic system and the orbito-frontal region.^57^^-^^60^

Opercular cortex: Seizures arising from opercular cortex and temporal lobe (temporo-opercular seizures) are characterized by the association of opercular semiology (unilateral or bilateral clonic, tonic or myoclonic jerk in the chin or perioral muscles, paresthesia in the oral cavity or in the perioral region, drooling anartria or motor aphasia)^12^^,^^61^^-^^67^ with some clinical, electrographic features of TLE in a patient with hippocampal sclerosis (unpublished data). Thompson et al.^12^ reported auditories auras in two patients with seizures arising from the opercular region, but this symptom is very hard to explain by opercular activation.

***Scalp-EEG findings in TL+ epilepsy***

Interictal and ictal recordings show some commons characteristic EEG patterns**:**

- Interictal, TL+ patients more frequently exhibit bilateral spikes and/or slow waves, as well as precentral (F4-C4; F3-C3) spike-and-waves complexes
- Ictally, the first EEG changes were more frequently localized over the anterior frontal (FP2-F4; FP1-F3) region, the temporoparietal (T5-P3; T6-P4) region and the precentral (F4-C4; F3-C3) region. These changes are found to be more frequently associated with the TF, TPO and TI subgroups, respectively.^5^ These findings are supported by some reports utilizing MEG.^16^

***PET studies*** ^14^^,^^15^

In a study of 54 consecutive patients with pharmacoresistant TLE that were retrospectively enrolled after a comprehensive presurgical evaluation (at least brain MRI, 18FDG-PET, surface video-EEG electroclinical exploration, and SEEG recordings), 18FDG-PET was especially used with MRI and surface video-EEG electroclinical exploration to guide SEEG recordings. The authors found that patients with TL+ epilepsy showed temporal hypometabolism ipsilateral to the atrophic hippocampus, involving the middle and superior temporal gyrus, the uncus and the PHG, but also one of the following structures: the lingual gyrus, the inferior parietal lobule and the supramarginal gyrus, the pre- and post-central gyrus, the inferior and middle frontal gyrus, the rectal gyrus or the insula. On the whole, the extratemporal cortical involvement was a typical finding for TL+ epilepsy.^14^

***Evoked related potentials in TL+ epilepsy***

The memory-related modulations of the N400 and P600 are usually referred to as evoke related potential “old/new” effects. These studies have demonstrated large amplitude gradients and local polarity reversals within and adjacent to the hippocampal formation and amygdala, suggesting a local generation of these potentials. Hippocampal recordings have revealed that both N400 and P600 effects were less pronounced or even absent in patients with TL+ epilepsy compared with patients with TLE.^68^^,^^69^

***Functional MRI (fMRI) in TL+ epilepsy***

Preliminary studies showed that magnetic source imaging (MSI) may be used to help identify the epileptogenic zone and epileptic networks including the temporal plus regions related with those networks. MSI may detect interictal spikes from mesial temporal structures, and therefore, may provide important localizing information in patients with mesial temporal sclerosis, especially when MRI and/or ictal scalp EEG are not localizing.^30^ An MSI study was performed on 22 patients with mesial temporal sclerosis candidates for surgical treatment. 60% of patients with non-localizing ictal scalp EEG had well-localized spikes on MSI ipsilateral to the side of surgery and 66.7% of patients with non-localizing MRI had well-localized spikes on MSI ipsilateral to the side of surgery.^31^

Continuous EEG-fMRI is a simple neuroimaging tool that has already improved initial presurgical planning by helping to identify irritative foci that necessitate further study with more invasive techniques to identify the epileptogenic region. Notwithstanding these advantages, an early study reported that continuous EEG-fMRI yields low sensitivity in definitively identifying an irritative focus in the mesial temporal lobe.^32^ Although this study made no distinction between patients whose EEG showed slow and high firing, a recent study suggests that firing rates could have a major role in detecting brain hemodynamic activation related to interictal epileptiform discharges.^33^

The study of Avesani et al.^30^ provided information related with the possible EEG-fMRI patterns in temporal epilepsy, however, the authors did not explore the differences between patients with temporal and temporal plus epilepsies. This study sought more information on blood oxygen level dependent activation, especially contralateral temporal and extratemporal spread, during continuous EEG-fMRI recordings in four patients with mesial temporal sclerosis. fMRI analysis confirmed a single activation in the mesial temporal region in two patients whose EEG showed unilateral focal activity, while it demonstrated a bilateral activation in the mesial temporal regions in the other two patients. In the third patient (with the most drug-resistant form and also extratemporal clinical signs), fMRI demonstrated an activation in the supplementary motor area, ipsilateral to the irritative focus. This study confirms the most significant activation with a high firing rate of the irritative focus, but also suggests the importance of using new techniques (such as EEG-fMRI to examine cerebral blood flow) to identify the contralateral limbic activation, and any other extratemporal activations, possible causes of drug resistance in temporal plus epilepsies that may require a more precise presurgical evaluation with invasive techniques.

***Invasive coverage of temporal and “plus” cortices-The planning***

As the diagnosis of TL+ epilepsy relies on invasive recordings, it is very important to get an adequate spatial intracerebral sampling of temporal and extratemporal structures.^13^ Structures that should be sampling include:

- Temporal lobe: amygdala, hippocampus, parahippocampus, EC, superior, medium and inferior temporal gyrus and temporopolar region
- Extratemporal structures: it is important to take into account the possible hypothesis (temporo-insular epilepsy, TPO epilepsy, temporo-perisylvian epilepsy, temporo-frontal epilepsy (temporo-opercular-frontal or temporo-fronto-basal).

Electrodes for the insular cortex should be inserted using lateral orthogonal trajectory through the fronto-parietal and temporal operculum cortex or using an oblique trajectory through frontal or parietal cortices.

When orbito-temporal epilepsy is suspected investigation of gyrus rectus, orbital cortex, frontopolar cortex and anterior cingulated cortex and temporal lobe are needed. 

In the cases of TPO epilepsy the targets should include the lingual lobule, fusiform gyrus, the precuneus and posterior cingulate cortex, the angular and supramarginal gyri, the temporal lobe and an electrode to register the parahippocampus electrical activity. These implantation patterns, obviously, depend on the knowledge we have, at the time of implantation, of the anatomo-functional systems that underlie the electroclinical features of TL+ seizures, so they can change with time.

In temporo-perisylvian epilepsies almost eight electrodes should be inserted in the temporal lobe including amygdala, hippocampus (three electrodes: anterior, middle and posterior), EC, temporopolar cortex, medium, and inferior temporal gyrus. It is also important to cover the perisylvian cortex: angular, supramarginal gyri, all upper blank of the operculum (anterior, medium and posterior operculum), and three electrodes should be placed in the superior temporal gyrus (anterior, medium and posterior).^13^.

## Conclusion

The TL+ epilepsy is a diagnosis that can be done only after the invasive recordings have been analyzed verifying that the epileptogenic network encompasses the temporal lobe and some of the neighboring cortical areas. However, an adequate suspicion may arise based on clinical, EEG and imaging data.

## Notes:

**How to cite this article:** Andrade-Machado R, Benjumea-Cuartas V. Temporal plus epilepsy: Anatomo-electroclinical subtypes. Iran J Neurol 2016; 15(3): 153-63.
